# Probiotics and Prebiotics in Pediatrics: What Is New?

**DOI:** 10.3390/nu11020431

**Published:** 2019-02-19

**Authors:** Yvan Vandenplas, Francesco Savino

**Affiliations:** 1 KidZ Health Castle, UZ Brussel, Vrije Universiteit Brussel, 1090 Brussels, Belgium; 2 Department of Pediatrics, Ospedale Infantile Regina Margherita, Azienda Ospedaliera, Universitaria Città della Salute e della Scienza di Torino, Piazza Polonia, 94, 10126 Turin, Italy; francesco.savino@unito.it

Probiotics and prebiotics are a hot topic in pediatric research. Human milk oligosaccharides have been recognized to enhance the development of a bifidogenic microbiome in infants. In this issue, many different clinical conditions are discussed in which probiotics and prebiotics can interfere with the microbiome. This editorial for a special issue of *Nutrients* contains 17 papers, a mixture of reviews and original research, reflecting the broad and evolving interest and researches in this topic, such as diarrhea, atopic diseases, infantile colic, celiac, necrotizing enterocolitis, constipation. However, in the pediatric age, manipulation of that microbiome still leads to inconclusive results as studies provide often contradictory data. The inconclusive data may be explained by the fact that dysbiosis is likely to be only one of several interfering factors causing these different conditions. In conclusion, the manuscripts in this issue raise a lot of aspects and questions and offer challenges for future research. 

The evolution of knowledge on this topic in recent years has allowed us to conclude that there is currently sufficient enough evidence to conclude that the role of the gastro-intestinal microbiome during the first month of life is crucial for a balanced development of the immune system. The interest in the human microbiome and its interplay with the host has exploded and provided new insights on its role in conferring host protection and regulating host physiology, including the correct development of immunity [1,2]. *Bifidobacterium breve* is the dominant species in the gut of breast-fed infants and it has also been isolated from human milk. It has antimicrobial activity against human pathogens, it does not possess transmissible antibiotic resistance traits, it is not cytotoxic and it has immuno-stimulating abilities [3]. Probiotic supplementation during pregnancy and in the neonatal period might reduce some maternal and neonatal adverse outcomes [4]. The current evidence on the efficacy of probiotics for the management of pediatric functional abdominal pain disorders, such as functional constipation, irritable bowel syndrome, functional abdominal pain is rather disappointing as no single strain, the combination of strains or synbiotics can be recommended for the management of these conditions [5]. 

Allergic individuals have a different microbiome than non-allergic. The “microbiota hypothesis” ties the increase in allergy rates observed in highly developed countries over the last decades to disturbances in the gut microbiota [6]. Diaz et al showed that infants with non-IgE mediated allergy have a different microbiome compared to healthy infants, while being on an elimination diet [7]. Moreover, the protein source (formula of vegetable origin, casein or whey hydrolysate) result in a different composition of the microbiome [7]. The clinical relevance of these findings needs to be further investigated. Lactobacillus (L.) administration might also be of interest in children with chronic immune disorders, such as asthma [8]. Results of a prospective, double blind, randomized Chinese study with four groups (*L. paracasei*, *L. fermentum*, their combination and placebo) showed lower asthma severity and better Childhood Asthma Control Test scores [8]. The group treated with both probiotics improved most, as increased peak expiratory flow rates and decreased IgE levels were shown [8]. Thus, lactobacillus administration, at least the strains tested, can contribute the clinical improvement in children with asthma [8]. A meta-analysis showed that *L. rhamnosus GG* was ineffective in the reduction of atopic dermatitis [9]. 

Infantile colic is a common condition, occurring in about 20 % of all infants, of unknown pathogenesis that causes frustration and anxiousness in families, which then seek effective management [10]. Dysbiosis and chronic inflammation are likely to be part of the pathophysiologic mechanisms of infantile colic [11]. A study from Ukraine showed that a combination of *L. rhamnosus* 19070-2 and *L. reuteri* and a small amount of a prebiotic, fructo-oligosaccharide (FOS), resulted in a significant decrease of crying time compared to the natural evolution in the placebo group [12]. These data confirm previous literature, mainly using *L. reuteri* alone, showing that lactobacilli decrease infantile colic in exclusively breastfed infants [13]. A probiotic mixture was also shown to reduce crying time in exclusively breastfed infants compared to placebo, although no differences between the groups were found regarding anthropometric data, bowel movements, stool consistency or microbiota composition [14]. Unfortunately, data on the outcome of probiotic administration in formula fed infants presenting with infantile colic are still missing. *L. reuteri* DSM 17938 may be considered for the management of breastfed colic infants, while data on other probiotic strains, probiotic mixtures or synbiotics are limited in infantile colic [5].

The ESPGHAN working group on probiotics and prebiotics recommended considering the addition of some probiotic strains to oral rehydration therapy in the management of infants with acute gastroenteritis [15]. The additional benefit of *L. reuteri* DSM 17938 and zinc was evaluated compared to oral rehydration alone in a study, including 51 children with acute gastroenteritis [16]. Although there was a trend that the probiotic and zinc supplemented group did better, the outcome was not statistically significant better [16]. Two other large trials, with *L. rhamnosus GG* reported also a negative outcome [17,18]. *Bacillus clausii* was tested in six randomized controlled trials, including 1298 [19]. Data arising from the pooled analysis showed that *Bacillus clausii* significantly reduced the duration of diarrhea with a mean difference of -9.12 hours only compared with control. Stool frequency was not significantly different after *Bacillus clausii* administration compared with the control group [19]. A randomized trial in India with *Bacillus clausii* compared to placebo reported a statistically significant difference in duration of diarrhea of only six hours, with a difference of one defecation per day at day 4 [20]. These findings question the importance of the selection of patients, and the strain selection of the probiotic. Shortening of the duration of diarrhea might have been shown to be statistically reduced, but may lack clinical significance of benefit [19]. 

The use of probiotics among very low-birth-weight infants is constantly increasing, as probiotics are believed to reduce the incidence of severe diseases, such as necrotizing enterocolitis (NEC) and late-onset sepsis and to improve feeding tolerance [21]. According to feeding type, the beneficial effect of probiotics was confirmed only in exclusively human milk-fed preterm infants [22]. Fifty-one randomized controlled trials were included in a review by the ESPGHAN working group on pre- and probiotics, involving 11,231 preterm infants [23]. Most strains or combinations of strains were only studied in one or a few trails [23]. Only 3 of 25 studied probiotic treatment combinations showed a significant reduction in mortality rates [23]. Seven treatments reduced NEC incidence, two reduced late-onset sepsis, and three reduced time until full enteral feeding [23]. Among human milk fed infants, only probiotic mixtures, and not single-strain products, were effective in reducing late onset sepsis [22]. Human milk oligosaccharides (HMO) have a strong prebiotic effect, and stimulate the development of a bifidogenic microbiome in breastfed infants. 

HMOs may support immune function development and provide protection against infectious diseases directly through the interaction of the gut epithelial cells or indirectly through the modulation of the gut microbiota, including the stimulation of the bifidobacteria [24,25]. The limited clinical data suggest that the addition of HMOs to infant formula seems to be safe and well tolerated, inducing a normal growth and suggesting a trend towards health benefits [24]. Gut immaturity in preterm infants leads to difficulties in tolerating enteral feeding and bacterial colonization and high sensitivity to NEC, particularly when breast milk is insufficient [26]. The HMOs diversity and the levels of Lacto-N-difucohexaose I were found to be lower in samples from mothers of infants that developed NEC, as compared to non-NEC cases at all sampling time points [27]. Lacto-N-difucohexaose I is only produced by secretor and Lewis positive mothers. This is significant, but inconsistent with associations between 3’-sialyllactose and 6’-sialyllactose, and culture-proven sepsis; and consists of weak correlations between several HMOs and growth rate [27]. However, the benefit of HMO supplementation in preterm infants is debated [26]. These findings highlight once more that a priority research topic is the understanding why about 20% of the mothers are "non-secretors", since all data suggest that infants of secretor mothers have a better health outcome than there of non-secretors. 

Constipation is still a frequent functional gastro-intestinal disorder in infants, occurring in about 10 % [10]. In a Brazilian, randomized, placebo-controlled, double blind trial, fructo-oligosaccharides (FOS) or placebo was given at a dosage of 6, 9 or 12 g daily based on the infants’ weight groups of 6.0–8.9 kg, 9.0–11.9 kg or over 12.0 kg, respectively [28]. Therapeutic success occurred in 83.3% of the FOS group infants and in as much as 55.6% of the control group [28]. The placebo effect in this trial was very high, suggesting again that reassurance is the cornerstone of the management of functional disorders in infants. But, compared with the control group, the FOS group exhibited a higher frequency of softer stools and fewer episodes of straining and/or difficulty passing stools [28]. Further, after one month, the Bifidobacterium sp. count was higher in the FOS group [28].

Celiac disease is a chronic autoimmune enteropathy triggered by dietary gluten exposure in genetically predisposed individuals [2]. Despite ascertaining that gluten is the trigger in celiac disease, evidence has indicated that also intestinal microbiota is somehow involved in the pathogenesis, progression, and clinical presentation of the disease [2]. Patients with celiac disease have an increased abundance of *Bacteroides* spp. and a decrease in *Bifidobacterium* spp. [2]. A six-week multispecies probiotic treatment improved the severity of irritable bowel syndrom-type symptoms, in celiac patients on a strict glutenfree diet and was associated with a modification of gut microbiota, characterized by an increase of bifidobacteria [28]. The role of prebiotics in the nutritional management of chronic conditions, such as celiac disease in patients on a glutenfree diet is a different area of interest. Iron deficiency anemia occurs in up to almost half of the patients diagnosed with celiac disease. A randomised trial with an oligofructose enriched inulin administered during three months to celiac patients failed to show a clear benefit of a bifidogenic microbiome on nutritional (ferritin, hemoglobin) and inflammatory (C-reactive protein) parameters, although a decrease in hepcidin was shown [29]. Hepcidin is a key regulator of the entry of iron into the circulation and considered to be an interesting and useful marker. 

Different aspects of pro- and prebiotics in pediatrics are presented and discussed in this special issue. The overall conclusion suggests that although there is a physiologic and patho-physiological ground regarding the impact of a balanced microbiome on different health aspects in infants and children, clinical outcomes are often contradictory. Future research and trials must reveal relevant outcomes about which there is a consensus regarding. It should be mandatory to report the specific strains of probiotics. Studies should be done with commercial products. Therefore, further research on the impact of manipulation with probiotic and prebiotic of the gastrointestinal microbiome in pediatrics is still needed.

## References

[1] Dominguez-Bello M.G., Godoy-Vitorino F., Knight R., Blaser M.J. (2019). Role of the microbiome in human development. Gut.

[2] Cristofori F., Indrio F., Miniello V.L., De Angelis M., Francavilla R. (2018). Probiotics in celiac disease. Nutrients.

[3] Cionci N.B., Baffoni L., Gaggìa F., Di Gioia D. (2018). Therapeutic microbiology: The Role of Bifidobacterium breve as food supplement for the prevention/treatment of paediatric diseases. Nutrients.

[4] Baldassarre M.E., Palladino V., Amoruso A., Pindinelli S., Mastromarino P., Fanelli M., Di Mauro A., Laforgia N. (2018). Rationale of probiotic supplementation during pregnancy and neonatal period. Nutrients.

[5] Pärtty A., Rautava S., Kalliomäki M. (2018). Probiotics on pediatric functional gastrointestinal disorders. Nutrients.

[6] Cukrowska B. (2018). Microbial and nutritional programming—The importance of the microbiome and early exposure to potential food allergens in the development of allergies. Nutrients.

[7] Díaz M., Guadamuro L., Espinosa-Martos I., Mancabelli L., Jiménez S., Molinos-Norniella C., Pérez-Solis D., Milani C., Rodríguez J.M., Ventura M. (2018). Microbiota and derived parameters in fecal samples of infants with non-IgE cow’s milk protein allergy under a restricted diet. Nutrients.

[8] Huang C.F., Chie W.C., Wang I.J. (2018). Efficacy of Lactobacillus administration in school-age children with asthma: A randomized, placebo-controlled trial. Nutrients.

[9] Szajewska H., Horvath A. (2018). Lactobacillus rhamnosus GG in the primary prevention of eczema in children: A systematic review and meta-analysis. Nutrients.

[10] Vandenplas Y., Abkari A., Bellaiche M., Benninga M., Chouraqui J.P., Çokura F., Harb T., Hegar B., Lifschitz C., Ludwig T. (2015). Prevalence and health outcomes of functional gastrointestinal symptoms in infants from birth to 12 months of age. J. Pediatr. Gastroenterol. Nutr..

[11] Savino F., Tarasco V. (2010). New treatments for infant colic. Curr. Opin. Pediatr..

[12] Gerasimov S., Gantzel J., Dementieva N., Schevchenko O., Tsitsura O., Guta N., Bobyk V., Kaprus V. (2018). Role of Lactobacillus rhamnosus (FloraActive™) 19070-2 and Lactobacillus reuteri (FloraActive™) 12246 in infant colic: A randomized dietary study. Nutrients.

[13] Sung V., D’Amico F., Cabana M.D., Chau K., Koren G., Savino F., Szajewska H., Deshpande G., Dupont C., Indrio F. (2018). Lactobacillus reuteri to treat infant colic: A meta-analysis. Pediatrics.

[14] Baldassarre M.E., Di Mauro A., Tafuri S., Rizzo V., Gallone M.S., Mastromarino P., Capobianco D., Laghi L., Zhu C., Capozza M. (2018). Effectiveness and safety of a probiotic-mixture for the treatment of infantile colic: A double-blind, randomized, placebo-controlled clinical trial with fecal real-time PCR and NMR-based metabolomics analysis. Nutrients.

[15] Lo Vecchio A., Vandenplas Y., Benninga M., Broekaert I., Falconer J., Gottrand F., Lifschitz C., Lionetti P., Orel R., Papadopoulou A. (2016). An international consensus report on a new algorithm for the management of infant diarrhoea. Acta Paediatr..

[16] Maragkoudaki M., Chouliaras G., Moutafi A., Thomas A., Orfanakou A., Papadopoulou A. (2018). Efficacy of an oral rehydration solution enriched with Lactobacillus reuteri DSM 17938 and zinc in the management of acute diarrhoea in infants: A randomized, double-blind, placebo-controlled trial. Nutrients.

[17] Freedman S.B., Williamson-Urquhart S., Farion K.J., Gouin S., Willan A.R., Poonai N., Hurley K., Sherman P.M., Finkelstein Y., PERC PROGUT Trial Group (2018). Multicenter trial of a combination probiotic for children with gastroenteritis. N. Engl. J. Med..

[18] Schnadower D., Tarr P.I., Casper T.C., Gorelick M.H., Dean J.M., O’Connell K.J., Mahajan P., Levine A.C., Bhatt S.R., Roskind C.G. (2018). Lactobacillus rhamnosus GG versus placebo for acute gastroenteritis in children. N. Engl. J. Med..

[19] Ianiro G., Rizzatti G., Plomer M., Lopetuso L., Scaldaferri F., Franceschi F., Cammarota G., Gasbarrini A. (2018). Bacillus clausii for the treatment of acute diarrhea in children: A systematic review and meta-analysis of randomized controlled trials. Nutrients.

[20] Sudha M.R., Jayanthi N., Pandey D.C., Verma A.K. (2019). Bacillus clausii UBBC-07 reduces severity of diarrhoea in children under 5 years of age: A double blind placebo controlled study. Benef. Microbes.

[21] Aceti A., Beghetti I., Maggio L., Martini S., Faldella G., Corvaglia L. (2018). Filling the Gaps: Current Research Directions for a Rational Use of Probiotics in Preterm Infants. Nutrients.

[22] Aceti A., Maggio L., Beghetti I., Gori D., Barone G., Callegari M.L., Fantini M.P., Indrio F., Meneghin F., Italian Society of Neonatology (2017). Probiotics prevent late-onset sepsis in human milk-fed, very low birth weight preterm infants: Systematic review and meta-analysis. Nutrients.

[23] Van den Akker C.H.P., van Goudoever J.B., Szajewska H., Embleton N.D., Hojsak I., Reid D., Shamir R., ESPGHAN Working Group for Probiotics, Prebiotics & Committee on Nutrition (2018). Probiotics for preterm infants: A strain-specific systematic review and network meta-analysis. J. Pediatr. Gastroenterol. Nutr..

[24] Vandenplas Y., Berger B., Carnielli V.P., Ksiazyk J., Lagström H., Sanchez Luna M., Migacheva N., Mosselmans J.M., Picaud J.C., Possner M. (2018). Human milk oligosaccharides: 2′-Fucosyllactose (2′-FL) and Lacto-N-Neotetraose (LNnT) in infant formula. Nutrients.

[25] Triantis V., Bode L., van Neerven R.J.J. (2018). Immunological effects of human milk oligosaccharides. Front. Pediatr..

[26] Bering S.B. (2018). Human milk oligosaccharides to prevent gut dysfunction and necrotizing enterocolitis in preterm neonates. Nutrients.

[27] Wejryd E., Martí M., Marchini G., Werme A., Jonsson B., Landberg E., Abrahamsson T.R. (2018). Low diversity of human milk oligosaccharides is associated with necrotising enterocolitis in extremely low birth weight infants. Nutrients.

[28] Souza D.D.S., Tahan S., Weber T.K., Araujo-Filho H.B., de Morais M.B. (2018). Randomized, double-blind, placebo-controlled parallel clinical trial assessing the effect of fructooligosaccharides in infants with constipation. Nutrients.

[29] Feruś K., Drabińska N., Krupa-Kozak U., Jarocka-Cyrta E. (2018). A randomized, placebo-controlled, pilot clinical trial to evaluate the effect of supplementation with prebiotic Synergy 1 on iron homeostasis in children and adolescents with celiac disease treated with a gluten-free diet. Nutrients.

